# I Am, I Am, I Am: Seventeen Brushes With Death, by Maggie O’Farrell

**DOI:** 10.1136/practneurol-2019-002310

**Published:** 2019-07-04

**Authors:** Tom J Moullaali, Shona Scott

**Affiliations:** 1 Centre for Clinical Brain Sciences, University of Edinburgh, Edinburgh, UK; 2 Department of Clinical Neurology, Western General Hospital, Edinburgh, UK

**Keywords:** cerebellar ataxia, vertigo, speech, articulation, paediatric neurology

The Edinburgh Neurology Book Club had a unique opportunity to explore Maggie O’Farrell’s^1^ memoirs ‘I Am, I Am, I Am: Seventeen Brushes With Death’ with the author present, which provided an intimate forum for reflection on the common ground we shared: an unusual familiarity with death. O’Farrell suffered a mysterious neurological illness in childhood—we settled on acute cerebellitis, but the case wasn’t closed—which was a focal point and catalyst for her unsettlingly frequent encounters with death in childhood and early adulthood. Chapters purposefully meander through time and take their titles from the body part at risk, opening with ‘Neck (1990)’, a chilling recount of a near miss with a murderous predator who claimed the life of another young female traveller only days later. In ‘Lungs (1988)’, her impulsive younger-self jumps into deep water and becomes increasingly disoriented and panicked when the route to the surface evades her vulnerable proprioceptive powers. She concludes with ‘Daughter (The present day)’, which O’Farrell explained was the hardest for her to tell—her child’s extreme nut allergy resulted in countless episodes of anaphylaxis in her early life, and as a result, frequent and varied experience of emergency medical care.

O’Farrell’s memoirs provided an intriguing substrate for discussion: her neurological illness and her description of its effect on all aspects of her life; her depiction of doctors and their key role in her most formative experiences (childhood and motherhood) and finally, her familiarity in the face of death, and how this shaped her. In the run-up to this book club, there was a healthy mix of admiration for O’Farrell’s literary splendour (a refreshing respite from bland scientific text!), and cynicism about some of her more tenuous ‘brushes’ with death. However, with the author present, critical discussion—particularly interrogation of the finer details of her neurological diagnosis—was tempered. Instead, we drew inspiration from O’Farrell’s willingness to share her personal stories with us, both through her writing, and now in person.


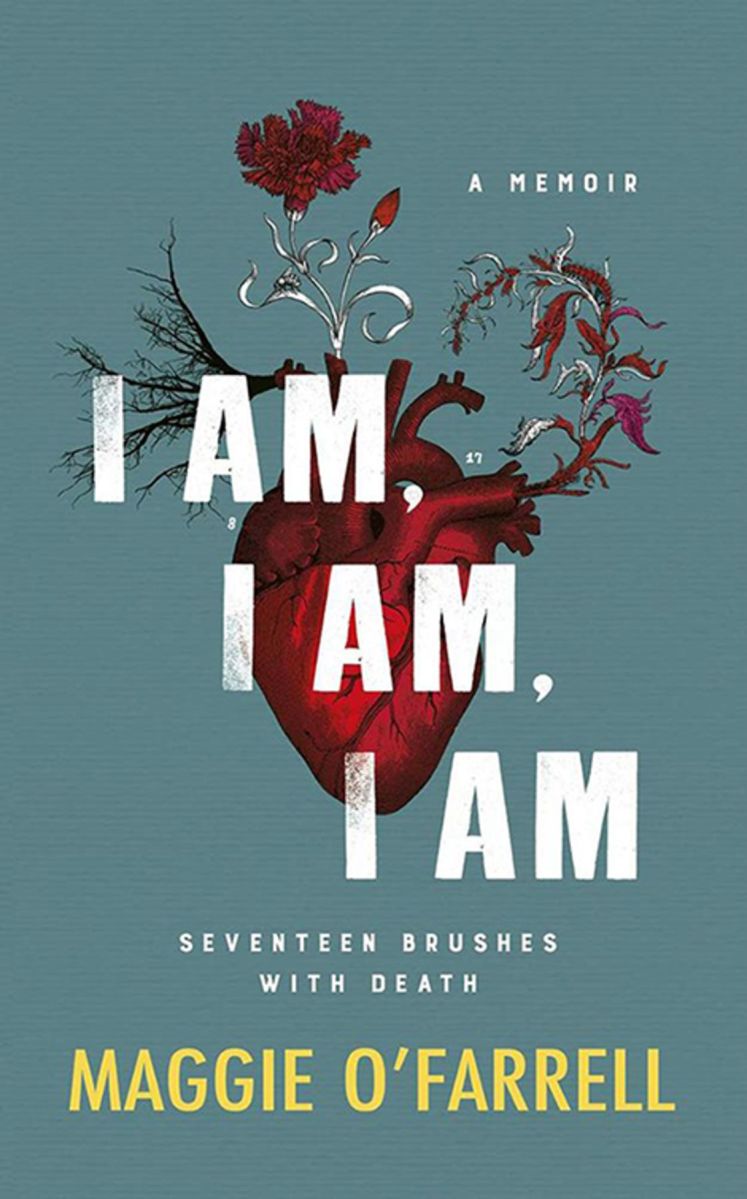


In our early exchanges, O’Farrell was an observer. The conversation started where we are conditioned to be comfortable: discussion of medicalised experiences through the distorted and dispassionate doctor’s lens. We shared professional experiences with the typical detachment that serves as a coping mechanism to ensure day-to-day maintenance of our sanity: challenging consultations in the clinic and attending cardiac arrest calls where our best efforts were in vain. Our readiness to question our own approaches, our explanations, and the parts we play in the real-life emotional dramas unfolding in every day practice was varied. But O’Farrell’s presence, and her willingness and ability to describe vividly her personal experiences—many of which she associated with deep emotional pain—encouraged a rare openness and the discussion slowly softened. We began to share our own painful personal stories with O’Farrell, who admitted her book’s impact on others continued to surprise her.

As we allowed our vulnerabilities to surface, the conversation came full circle with renewed insight and empathy, and we closed by asking O’Farrell what she valued most in a doctor. Together we concluded that our patients require us first to make the correct diagnosis and to deliver appropriate interventions, but by retaining even a glimmer of insight into the human story of ill health and death, we cling to the shared ground that allows us to connect with each other during times of extreme vulnerability.
